# Functional Dissection of *Caenorhabditis elegans* CLK-2/TEL2 Cell Cycle Defects during Embryogenesis and Germline Development

**DOI:** 10.1371/journal.pgen.1000451

**Published:** 2009-04-10

**Authors:** Sandra C. Moser, Sophie von Elsner, Ingo Büssing, Arno Alpi, Ralf Schnabel, Anton Gartner

**Affiliations:** 1 Wellcome Trust Centre for Gene Regulation and Expression, College of Life Sciences, University of Dundee, Dundee, Scotland, United Kingdom; 2 Developmental Genetics, Technische Universität Braunschweig, Braunschweig, Germany; National Institute of Diabetes and Digestive and Kidney Diseases, United States of America

## Abstract

CLK-2/TEL2 is essential for viability from yeasts to vertebrates, but its essential functions remain ill defined. CLK-2/TEL2 was initially implicated in telomere length regulation in budding yeast, but work in *Caenorhabditis elegans* has uncovered a function in DNA damage response signalling. Subsequently, DNA damage signalling defects associated with CLK-2/TEL2 have been confirmed in yeast and human cells. The CLK-2/TEL2 interaction with the ATM and ATR DNA damage sensor kinases and its requirement for their stability led to the proposal that CLK-2/TEL2 mutants might phenocopy ATM and/or ATR depletion. We use *C. elegans* to dissect developmental and cell cycle related roles of CLK-2. Temperature sensitive (ts) *clk-2* mutants accumulate genomic instability and show a delay of embryonic cell cycle timing. This delay partially depends on the worm p53 homolog CEP-1 and is rescued by co-depletion of the DNA replication checkpoint proteins ATL-1 (*C. elegans* ATR) and CHK-1. In addition, *clk-2* ts mutants show a spindle orientation defect in the eight cell stages that lead to major cell fate transitions. *clk-2* deletion worms progress through embryogenesis and larval development by maternal rescue but become sterile and halt germ cell cycle progression. Unlike ATL-1 depleted germ cells, *clk-2*–null germ cells do not accumulate DNA double-strand breaks. Rather, *clk-2* mutant germ cells arrest with duplicated centrosomes but without mitotic spindles in an early prophase like stage. This germ cell cycle arrest does not depend on *cep-1*, the DNA replication, or the spindle checkpoint. Our analysis shows that CLK-2 depletion does not phenocopy PIKK kinase depletion. Rather, we implicate CLK-2 in multiple developmental and cell cycle related processes and show that CLK-2 and ATR have antagonising functions during early *C. elegan*s embryonic development.

## Author Summary

PI3K-related protein kinases (PIKKs) ATM and ATR are essential upstream components of DNA damage signalling pathways, while TOR-1 acts as a nutrient sensor. CLK-2/TEL2 is a conserved gene initially implicated in budding yeast telomere length regulation and uncovered in the same genetic screen as the yeast TEL1 ATM like kinase. CLK-2/TEL2 was first implicated in DNA damage response signalling by *C. elegans* genetics, a function confirmed in yeast and human cells. In addition, CLK-2/TEL2 is essential for cellular and organismal survival from yeasts to vertebrates, but the essential phenotypes were not defined. A direct interaction between CLK-2/TEL2 and all PI3K-related protein kinases and the reduction of PIKK protein levels upon CLK-2/TEL2 depletion lead to the widely discussed notion that CLK-2/TEL2 mutants might phenocopy PIKK depletion phenotypes. We take advantage of embryonic lineage analysis and germline cytology to dissect developmental and cell cycle related functions of CLK-2. CLK-2 depletion does not phenocopy PIKK kinase depletion. We rather link CLK-2 to multiple developmental and cell cycle related processes and show that CLK-2 and ATR have antagonising functions during early *C. elegan*s embryonic development. Furthermore, we implicate CLK-2 in a distinct cell lineage decision and show that its depletion leads to a novel germline cell cycle arrest phenotype.

## Introduction

CLK-2/TEL2 is a DNA damage checkpoint gene which is essential for viability in budding yeast, *C. elegans* and vertebrates. DNA damage checkpoints are essential for maintaining genome stability in response to DNA damage and act by coordinating DNA repair and by triggering a transient cell cycle arrest, or apoptosis of affected cells. The loading of a pair of highly conserved PI3 kinase-related kinases (PIKKs), ATM and ATR, to sites of DNA damage acts at the apex of DNA damage response pathways [1]. These kinases have overlapping substrate specificity and phosphorylate multiple targets including the kinases Chk1 and Chk2 [2],[3]. The first *C. elegans clk-2* allele initially referred as *rad-5 (mn159)*, was isolated in a screen for *C. elegans* mutants hypersensitive for ionizing irradiation [4]. *C. elegans clk-2* temperature sensitive mutants are embryonic lethal at the restrictive temperature of 25°C [5]–[7]. However, the cause of this embryonic lethality is not known. At the “permissive temperature” of 20°C both known *clk-2* temperature sensitive alleles lead to a slow growth phenotype that is particularly evident in the *clk-2 (qm37)* allele, which also shows a reduction in cyclic behaviours such as pharyngeal pumping [5],[6]. Furthermore, both alleles are defective in various DNA damage responses including DNA damage-induced germ cell apoptosis and cell cycle arrest when propagated at 20°C [5],[6]. CLK-2/TEL2 has been implicated in S-phase regulation and DNA damage checkpoint responses in fission yeast [8],[9], and human CLK-2/TEL2 is required for the DNA replication checkpoint and for DNA crosslink repair [10]. Human and yeast CLK-2/TEL2 directly bind to all PI3K-related protein kinases (PIKKs) and are considered to be required for maintaining their stability [8],[9].

Here we use the *C. elegans* experimental system to assess the essential functions of CLK-2 during development and cell cycle control. In worms cell cycle progression in early embryos occurs very rapidly, with alternating M and S phases and an apparent lack of gap phases [11]. The timing and pattern of cell division and differentiation is invariant and has been fully characterized [12]. Aberrant embryonic development can therefore be traced by cell lineage analysis and resolved at a cellular level [13]. A relatively high level of DNA damage is tolerated during rapid embryonic cell divisions, possibly as a result of natural selection that favours a rapid pace of replication at the expense of genome integrity [14]. Only high levels of DNA damage or replication failure lead to a DNA damage checkpoint-dependent slowing of cell cycle progression [14]. Interestingly, the DNA damage checkpoint is used during early embryogenesis to contribute to the asymmetry of the first zygotic cell division [15]. In contrast to this, cell proliferation is much slower in the *C. elegans* germline and DNA damage checkpoint signalling is much more sensitive [11]. The germline is the only proliferative tissue in adult worms. The gonad contains various germ cell types that are arranged in an ordered distal to proximal gradient of differentiation [16],[17]. The distal end of the gonad is comprised of a mitotic stem cell compartment, which is followed by the transition zone where entry into meiotic prophase occurs. Proximal to the transition zone most germ cells are in meiotic pachytene and subsequently complete meiosis and concomitantly undergoing oogenesis in the proximal gonad. DNA replication failure and DNA double strand breaks lead to a prolonged cell cycle arrest of mitotic germ cells and to apoptosis of meiotic pachytene germ cells [18]. In this DNA damage response pathway CLK-2 and ATL-1 act as upstream DNA damage signalling molecules, while the worm p53 like gene *cep-1* is only required for apoptosis [19],[20]. Thus, CLK-2 and ATL-1 are part of sensitive germ cell DNA damage checkpoint pathways that ensure the faithful transmission of genetic information from one worm generation to the next.

*C. elegans clk-2* ts mutants show that CLK-2 is required for embryonic development [6],[7]. As these mutants show an increased level of DNA damage in germ cells at the restrictive temperature, the embryonic lethality might be caused by the accumulation of DNA damage that ultimately may result in the death of the embryo [21]. Given that ATR stability depends on CLK-2 [8],[9], the depletion of CLK-2 might phenocopy the *atl-1* (worm ATR) mutant phenotype, which is germline sterility associated with massive levels of DNA double strand breaks [22]. Furthermore, given that CLK-2 is required for the stability of all PIKKs *clk-2* mutations might mimic the phenotype of depleting other PIKKs such as TOR-1, implicated in nutrient sensing [23] and SMG1, a kinase involved in nonsense-mediated mRNA decay [24]. Finally, loss of CLK-2 function might result in distinct developmental defects not directly predicted from failing to maintain normal levels of PIKKs or from potential DNA replication and/or DNA damage signalling defects. In this study, we assess the essential defects associated with *clk-2* by analysing embryonic cell divisions by cell lineage analysis and by exploiting the *C. elegans* germline system. We show that *clk-2* mutants exhibit defects in early embryonic development and in germline cell cycle progression. These phenotypes do not overlap with reported *C. elegans* PIKK deletion phenotypes.

## Results

### Embryonic Cell Cycle Delay in *clk-2* Mutants Depends on *atl-1/chk-1*

**Figure 1:**
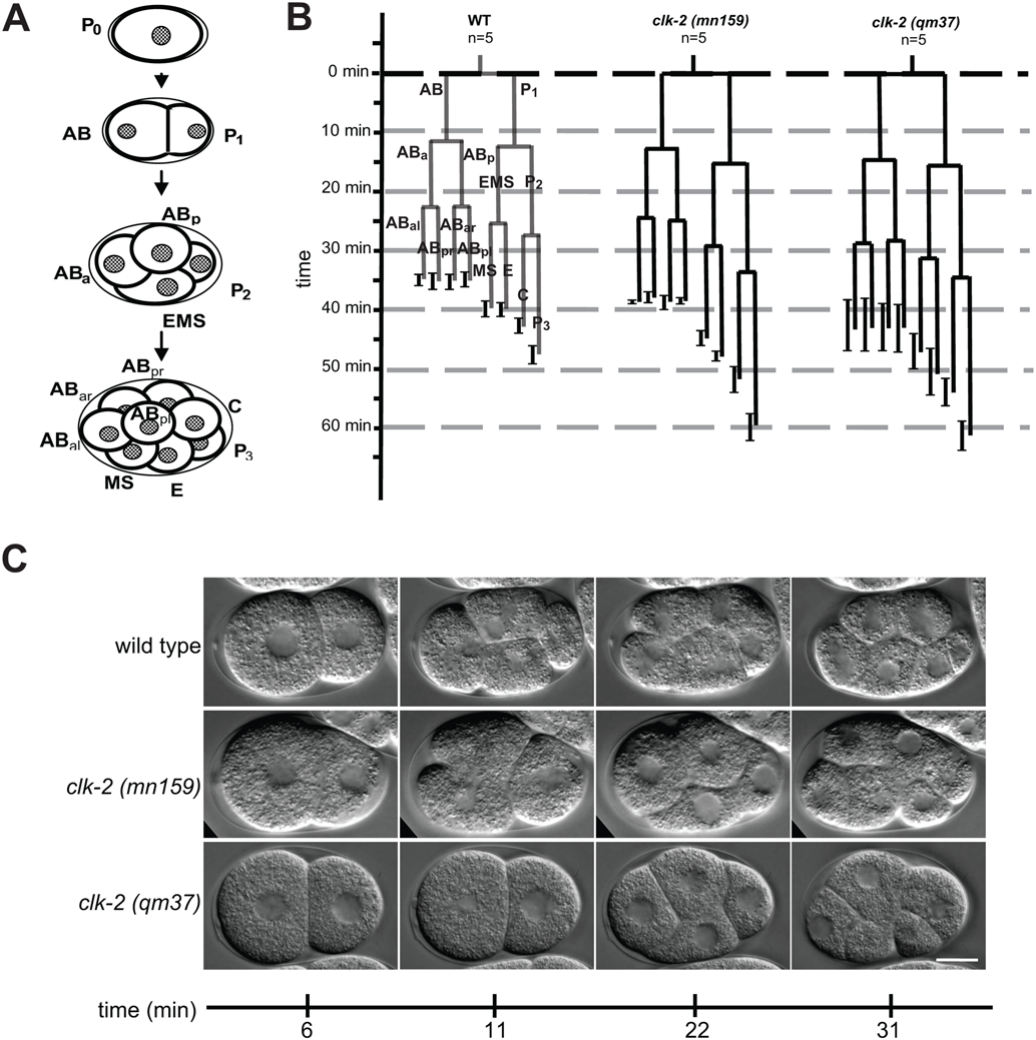
Delayed cell divisions in *clk-2* mutant embryos. A) Diagram of early *C. elegans* development. The newly formed *C. elegans* zygote undergoes a sequence of asymmetric and asynchronous cell divisions to produce the six founder cells called AB, MS, E, C, D and P4 [12],[58]. The first cleavage gives rise to the larger anterior founder cell AB and the smaller posterior cell P1. The AB cell begins a second symmetric cleavage, followed by the cleavage of P1 to produce EMS and P2. The division of EMS produces the E and the slightly larger MS founder cells. Shortly after the division of EMS, P2 divides to give rise to the C founder cell and P3. B) Lineage analysis of wild type and *clk-2* embryos. Horizontal lines indicate the time of cell division, while the vertical lines indicate the duration of the cell cycle for each blastomere during the first, second and third rounds of embryonic cell divisions. The names of the individual cells are indicated in the wild type panel. 0 min corresponds to the end of the P0 cytokinesis and is indicated by the horizontal dashed, black line. Average cell cycle times of five embryos are shown. Error bars indicate standard error of the mean (SEM) of accumulated cell cycle times. The cell cycle timing of individual cells is shown in Table S1. C) Nomarski images of early wild type and *clk-2* embryos undergoing the second and third round of mitotic divisions. All embryos are shown with anterior to the left and dorsal up. Scale bar: 10 µm.

**Figure 2:**
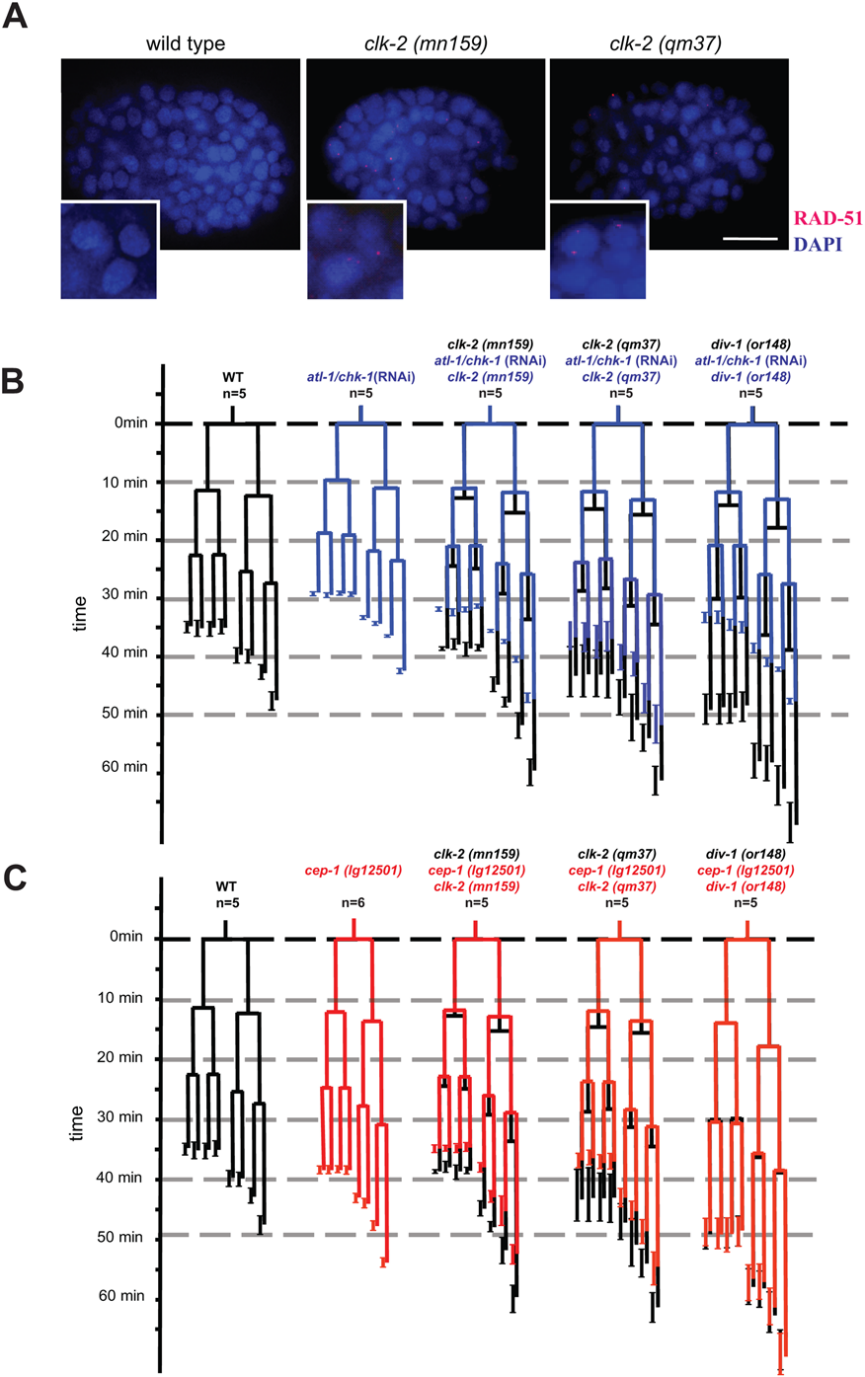
DNA double strand break accumulation in *clk-2* worms and attenuation of *clk-2* cell cycle delay by *atl-1/chk-1* (RNAi) and *cep-1*. A) Wild type and *clk-2* embryos were stained with anti RAD-51 antibodies (red) and DAPI (blue). Scale bar: 10 µm. B) Cell cycle timing is advanced in *atl-1/chk-1* (RNAi) embryos (blue) and *atl-1/chk-1* (RNAi) suppresses the cell cycle delay associated with *clk-2* and *div-1* mutants. C) The *cep-1 (lg12501)* deletion partially rescues the *clk-2* cell cycle delay but does not rescue the *div-1* cell cycle delay.

We wished to determine why *clk-2* mutants fail to complete embryogenesis. We therefore started our analysis by following the embryonic development of the two available recessive *clk-2* thermosensitive (ts) mutants, *mn159* and *qm37*, (Figure S1) by cell lineage analysis using 4D microscopy. Analysis of *clk-2* mutant lineages at the restrictive temperature of 25°C revealed that asymmetric cell divisions occurred normally during the first three embryonic cell cycles as previously reported [5],[6] but that cell division timing of all cells was delayed compared to wild type (Figure 1A, B, Table S1). This delay was more pronounced in *clk-2 (qm37)* than in *clk-2 (mn159)* (Figure 1B, Table S1). In the depicted recordings, the wild type embryo is at the 4-cell stage 11 min after cytokinesis of the P0 cell while the *clk-2 (mn159)* embryo is about to reach the three cell stage with the AB cell approaching cytokinesis (Figure 1C). The depicted *clk-2 (qm37)* embryo is at the two cell stage with the AB blastomere just having undergone nuclear envelope breakdown (Figure 1C). Thirty-one minutes after P0 cytokinesis wild type embryos are at the 8-cell stage while both *clk-2* mutants are in the 6-cell stage. We next aimed to determine the cause of the cell cycle delay associated with *clk-2* mutants. Given that *clk-2 (mn159)* worms show increased DNA double strand breaks in the mitotic zone of the adult *C. elegans* germline at the restrictive temperature [22], we reasoned that the cell cycle delay in *clk-2 (mn159)* and *(qm37)* embryos might be due to excessive DNA damage, potentially resulting from compromised DNA replication. We therefore tested whether RAD-51 foci, which are indicative of processed DNA double strand breaks or stalled replication forks [25], accumulate in *clk-2* embryos at the restrictive temperature. We indeed observed increased levels of RAD-51 foci in embryos examined between the 100 and 200 cell stage in both *clk-2 (mn159)* (2.14±0.62 foci/nucleus n = 7 embryos) and *clk-2 (qm37)* (0.97±0.19 foci/nucleus n = 8) mutants compared to wild type (0.2±0.02 foci/nucleus n = 6) (Figure 2A). These results indicate that *clk-2* mutants display a delay in embryonic cell cycle timing and increased genomic instability.

Given the delay in cell division timing and the accumulation of RAD-51 foci in *clk-2* mutants, we asked if the delay is due to the activation of the DNA replication checkpoint. Previous studies showed that the ATL-1/CHK-1 checkpoint is needed for sensing replication failure in *C. elegans* embryos [15]. Furthermore, the ATL-1/CHK-1 checkpoint contributes to developmental asymmetry by being in part responsible for the DNA replication delay in the P1 cell. Co-depletion of *atl-1* and *chk-1* is needed to fully inactivate the DNA replication checkpoint [15]. We observed that upon *atl-1/chk-1* depletion cell cycle timing is faster beyond the first embryonic cell division (Figure 2B, Table S1). We therefore conclude that the ATL-1/CHK-1 pathway acts in normal *C. elegans* early embryonic development to slow down cell cycle progression. As expected, *atl-1/chk-1* (RNAi) rescued the prolonged cell cycle delay associated with depleting the DIV-1 DNA polymerase primase alpha-subunit [15] (Figure 2B, Table S1). Importantly, RNAi-mediated *atl-1/chk-1* depletion largely rescued the delay in cell division timing associated with both *clk-2* mutants (Figure 2B, Table S1). Our results thus indicate that *clk-2 (mn159)* and *clk-2 (qm37)* mutations result in increased DNA damage, which triggers the ATL-1/CHK-1 checkpoint.

It has previously been shown that embryonic lethality associated with *dut-1* (RNAi), which leads to the misincorporation of uracil during DNA replication is partially rescued by *clk-2* (RNAi) and *chk-1* (RNAi) as well as by the *clk-2 (mn159)* mutation [26]. These results hint towards a checkpoint function of CLK-2 in embryonic cell divisions. We therefore assessed if CLK-2 functions in DNA damage checkpoint signalling in embryos and asked if the cell cycle delay caused by *div-1* (RNAi) depends on *clk-2*. We found that the delay in S-phase progression of the P1 cell caused by *div-1* (RNAi) is partially rescued by both *clk-2* ts alleles (Table S2). These results suggest that CLK-2 has a checkpoint function in early embryos. However, the AB cell cycle delay is not rescued likely due to the above described cell cycle delay associated with *clk-2* ts mutations.

### The Cell Cycle Delay of *clk-2* Mutants Depends on *cep-1*

It was reported that CLK-2 and CEP-1, the single *C. elegans* p53 homolog, cooperate in pathways leading to germ cell apoptosis upon treatment with ionizing irradiation (IR) [19],[20]. *cep-1* mutants are defective in IR induced apoptosis but are wild type for IR induced cell cycle arrest and DNA repair suggesting that CEP-1 acts downstream of CLK-2 in the DNA damage response pathway. Derry et al. also observed that a *cep-1* deletion partially rescues the slow growth phenotype associated with *clk-2 (mn159)* and *clk-2 (qm37)* [27]. We first confirmed the reported partial rescue of the slow growth phenotype of *clk-2 (mn159)* and *(qm37)* by the *cep-1 (lg12501)* deletion (Figure S2A/B) [27].

**Figure 3:**
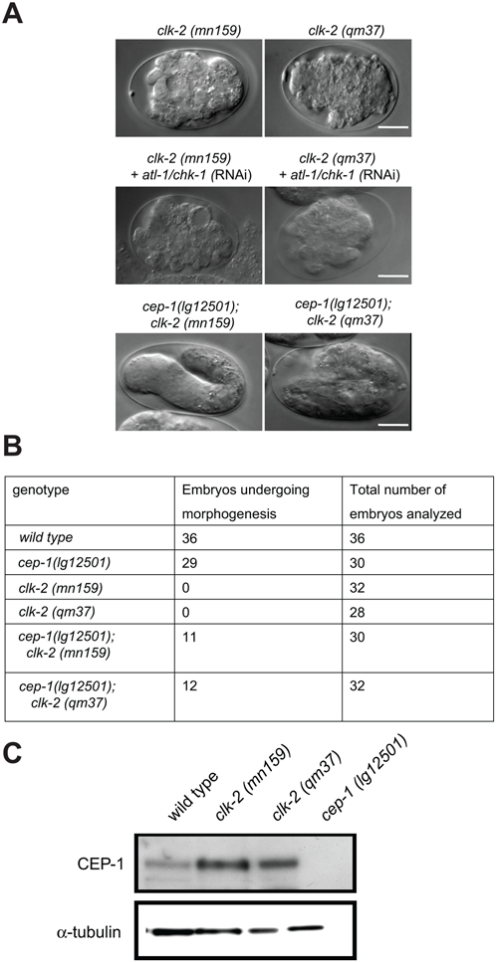
Genetic interactions between *cep-1* and *clk-2*. A) Representative *clk-2* and *clk-2 atl-1/chk-1* (RNAi) and *cep-1*; *clk-2* embryos. B) Ratio of embryos entering and arresting at the morphogenesis stage. C) Immunoblot of wild type and *clk-2* extracts with CEP-1 and α-tubulin antibodies. Lysates are from staged adult worms raised at 25°C.

Given the rescue of the *clk-2* slow growth phenotype by *cep-1* we wondered if *cep-1 (lg12501)* would suppress the embryonic cell cycle delay of *clk-2* mutants. *cep-1 (lg12501)*, which results in a slightly slower developmental rate compared to wild type, partially rescued the embryonic cell cycle delay associated with both *clk-2* alleles (Figure 2C, Table S1). In contrast, the cell cycle delay in *div-1* embryos was not rescued by *cep-1 (lg12501)* (Figure 2C, Table S1). This may indicate that distinct DNA lesions occurring in *clk-2* mutant embryos but not a general failure of DNA replication as it occurs in *div-1* mutations leads to the activation of a *cep-1* dependent checkpoint during early *C. elegans* embryogenesis. In addition, we found that *clk-2 (mn159)* or *(qm37)*; *cep-1 (lg12501)* double mutants develop to a later embryonic stage and often arrest in morphogenesis stage, with clear signs of tissue differentiation such as the formation of the pharynx or the appearance of gut granules. This late arrest never occurs in either *clk-2* single mutant or *atl-1/chk-1* (RNAi) depleted *clk-2* embryos (Figure 3A, B).

Given the rescue of the *clk-2* mutant cell cycle delay by a *cep-1* deletion, we asked if CEP-1 might be modified in *clk-2* mutant worms and assayed for changes in its abundance by western blotting. We found that the levels of CEP-1 protein were markedly increased in extracts prepared from synchronised adult *clk-2 (mn159)* and *clk-2 (qm37)* worms compared to wild type, indicating that the checkpoint triggered by *clk-2* mutations leads to the accumulation of CEP-1 (Figure 3C). This accumulation of CEP-1 likely results from increased CEP-1 in embryos. CEP-1 germline levels are not increased in *clk-2* mutants (data not shown) and besides embryonic and germline expression CEP-1 is only expressed in very few cells in the pharynx [19] (data not shown). In summary, we show that deleting *cep-1* partially rescues the slow growth phenotype associated with *clk-2* mutants and that CEP-1 accumulates in *clk-2* mutants. It will be interesting to determine the mechanism of CEP-1 accumulation and if other embryonic defects also lead to CEP-1 accumulation.

### A Spindle Orientation Defects Leads to Cell Fate Transformation of *clk-2* Mutants

**Figure 4:**
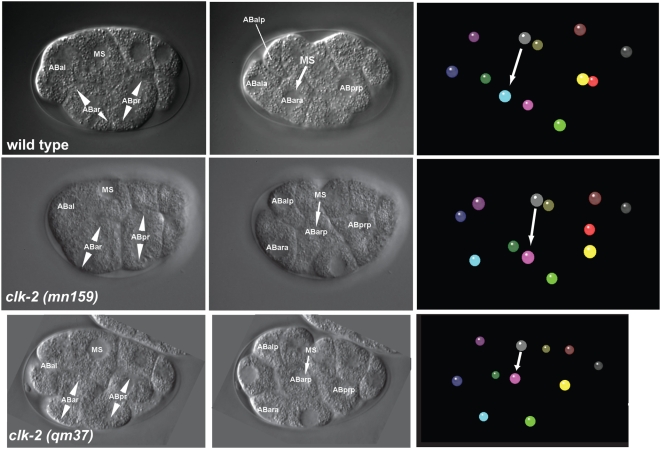
Lineage defects in *clk-2* mutant embryos. The left panels and middle panel show DIC images of representative focal planes of 8 and 12-cell stage wild type and *clk-2* embryos, respectively. The polarity of ABar and ABpr divisions is indicated by arrowheads. The right panel visualises the 12 embryonic cells in a 3D-model. The inductive signal from MS to ABara (in wild type) and ABarp (in *clk-2* mutants) is indicated by arrows in the middle and right panels. The grey ball represents the MS cell, turquoise represents ABara and pink ABarp. AB and MS are founder cells. a: anterior, p: posterior, l: left, r: right.

We speculated that there might also be phenotypes occurring in early *clk-2 (mn159)* and *clk-2 (qm37)* embryos that are not linked to the cell cycle delay of *clk-2* mutants. Indeed, our lineage analysis revealed that 2 out of 7 *clk-2 (mn159)* and 6 out of 12 *clk-2 (qm37)* mutant embryos recorded at 25°C exhibit a distinct lineage defect (Figure 4). We found an abnormal spindle rotation of the ABar cell (the anterior right granddaughter of the AB founder cell) at the 8-cell stage in *clk-2* mutants. In the six *clk-2 (qm37)* embryos showing the abnormal spindle rotation ABar divided on average 48±6° off the a-p axis placing ABarp towards the ventral side of the embryo. In five wild-type embryos ABar divided on average by 54±14° off the a-p axis placing ABarp towards the dorsal side of the embryo. The ABar spindle in the six affected *clk-2 (qm37)* embryos thus derived 102° from wild type. This abnormal rotation gives rise to mispositioned ABarp and ABara daughters at the 12 cell stage, bringing ABarp instead of ABara in contact to the MS blastomere (Figure 4, Videos S1, S2, and S3). The MS blastomere emits an inductive signal which in wild type is part of the left versus right cell fate decision (Figure 4, arrows, Videos S1, S2, and S3) [28]–[30]. As a consequence cell fates of the early founder cells are changed in the *clk-2* mutants, the ABara and ABarp blastomeres adopt the fates of their left counterparts, ABala and ABalp, respectively (data not shown). This change in cell fate identity leads to embryonic death. A failure of the ABar blastomere to rotate the spindle properly can be taken as an indication that spindles are generally not polarised properly [31], which is a hallmark of mutants in *mom-2* (wnt) and *mom-5* (frizzled) [32]. Future work will reveal, if *clk-2* influences the Wnt pathway directly or if the observed *clk-2* phenotype is independent of this pathway.

### *clk-2* Is Required for Germ Cell Proliferation

To further assess potential developmental and cell proliferation-associated defects of *clk-2* mutants, we analysed the germline of *clk-2* mutants. *clk-2* ts mutants are deficient in responding to DNA damaging agents [5] at the “permissive temperature” of 20°C and shifting *clk-2 (mn159)* mutants to 25°C at the L4 stage leads to the accumulation of DNA damage in affected germ cells [22]. However, these studies were done with the *clk-2* ts alleles. As it is not clear whether they act as null alleles at 25°C we analysed a *clk-2* deletion allele.

**Figure 5:**
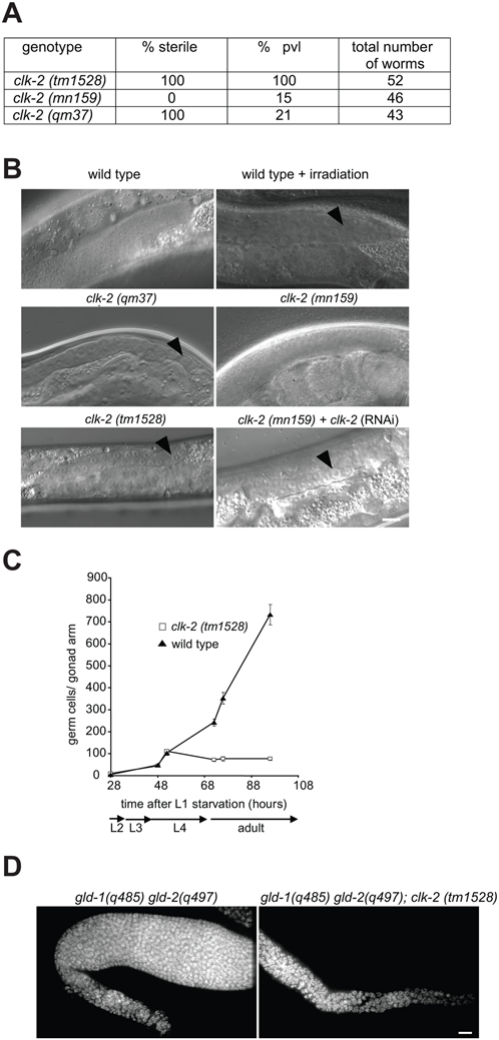
*clk-2* is required for germ cell proliferation. A) Fertility and Pvl phenotype of various *clk-2* mutants. *clk-2 (mn159)* and *(qm37)* were grown at 25°C, *clk-2 (tm1528)* was grown at 20°C. B) Nomarski DIC images of adult mitotic germlines. The distal tip cell is to the right of each germline. Arrowheads depict individual germ cell nuclei which are enlarged in c*lk-2 (tm1528)*, *clk-2 (qm37)* and in irradiated wild type animals. C) Number of germ cell nuclei per gonad arm in *clk-2 (tm1528)* (n = 10) and wild type (n = 10) worms. Worms were synchronised by L1 starvation, transferred to seeded NGM plates and grown at 20°C, fixed at indicated time points and stained with DAPI. Error bars represent SD. D). Images of *gld-2 (q497) gld-1 (q485)* (left) and *gld-2 (q497) gld-1 (q485)*; *clk-2 (tm1528)* (right) gonads stained with DAPI. Loss of CLK-2 results in a reduction of mitotic germ cells. Worms were grown at 20°C. Scale bar: 10 µm.

The *clk-2 (tm1528)* deletion allele provided by the Japanese *C. elegans* knockout consortium lacks part of the 5′ region, the first three exons, and a part of the fourth exon (Figure S1A). Western blotting with a CLK-2 specific antibody provided by Simon Boulton failed to detect any CLK-2 protein in *clk-2 (tm1528)* worm extracts (Figure S1B). We found that the major phenotype associated with the *clk-2 (tm1528)* deletion mutant kept at 20°C is not embryonic lethality but germline sterility (Figure 5A, see below) and that the same phenotype occurs when the *clk-2 (tm1528)* deletion mutant is kept at 25°C (data not shown). The *clk-2 (tm1528)* phenotype is recessive (data not shown). Given that *clk-2 (tm1528)* worms go through embryogenesis whereas *clk-2 (mn159)* and *(qm37)* worms arrest during embryogenesis at the restrictive temperature, we assume that *clk-2 (tm1528)* worms are rescued by maternal contribution. To ascertain that the missing embryonic lethality of the *clk-2 (tm1528)* mutant is indeed caused by the maternal supply we reviewed the phenotype of *clk-2 (mn159)* and *clk-2 (qm37)* worms by shifting those mutants to 25°C at the L1 stage. Under these conditions we found that *clk-2 (qm37)* worms are 100% sterile similar to *clk-2 (tm1528)* worms, while the weaker allele *mn159* does not lead to sterility (Figure 5A). Both ts alleles, as well as the deletion, lead to a protruding vulva phenotype (pvl) (Figure 5A). This phenotype is often associated with sterile germlines and general problems in postembryonic cell cycle progression [33]. *clk-2 (qm37)* and *clk-2 (tm1528)* gonades are significantly smaller in size than those of wild type and *clk-2 (mn159)* mutants and *clk-2 (qm37)* and *(tm1528)* gonads showed a dramatic reduction of germ cell numbers (Figure 5B, Figure S3). This reduction in germ cell numbers and germline sterility was also obtained upon *clk-2* RNAi in the weaker *clk-2 (mn159)* mutant, further indicating that the *clk-2 (qm37)* and *clk-2 (tm1528)* germline phenotypes represent the *clk-2* null phenotype (Figure 5B). These results are in contrast to a previous report which stated that no sterility of *clk-2 (qm37)* germlines was observed [6].

The reduced germ cell number raised the question whether CLK-2 is required for germ cell proliferation or germ cell differentiation. To address this question we performed a time course analysis of germline development in wild type and *clk-2 (tm1528)* worms. We found that both strains have similar numbers of germ cells up to the L4 stage at which point germlines of *clk-2 (tm1528)* worms stop proliferating (Figure 5C). To further assess if this phenotype is caused by a proliferation defect we took advantage of *gld-2 (q497) gld-1 (q485)* double mutants which have germlines that do not enter meiosis and are thus entirely proliferative. Comparing *gld-2 (q497) gld-1 (q485)* germlines to *gld-2 (q497) gld-1 (q485)*; *clk-2 (tm1528)* triple mutant germlines we found that germ cell numbers are dramatically reduced in the triple mutant indicating that *clk-2* has a role in germ cell proliferation rather than in germ cell differentiation (Figure 5D). In addition, *clk-2 (tm1528)* and *clk-2 (qm37)* germ cells are larger than wild type. This phenotype, which is reminiscent of arrested mitotic germ cells after ionizing irradiation, indicates that cells might stop cell division but continue with cellular growth [18] (Figure 5B, arrowheads). In summary, our data suggest that CLK-2 is required for cell cycle progression in germ cells.

### *clk-2* Germ Cells Arrest in an Early Prophase-Like Stage Independent of Checkpoint Activation

**Figure 6:**
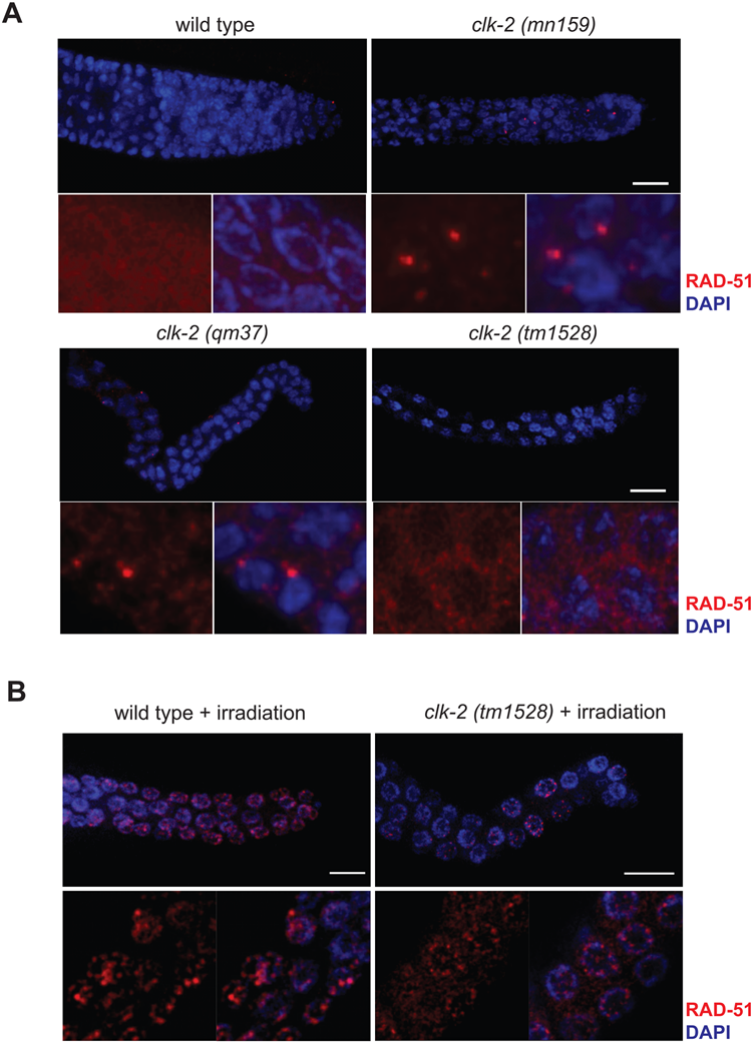
RAD-51 foci do not accumulate in *clk-2 (tm1528)* germlines. A) Representative images of fixed germlines from adult worms stained with anti-RAD-51 antibody (red) and DAPI (blue). Wild type and *clk-2 (tm1528)* were analysed after growth at 20°C, *clk-2 (mn159)* and *(qm37)* were analysed 24 hours after reaching the L4 stage upon shifting to 25°C at the L1 stage. Scale bar: 10 µm. Representative inserts depict mostly cytoplasmic RAD-51 staining in wild type and *clk-2 (tm1528)* germlines. B) RAD-51 nuclear foci formation in wild type and *clk-2 (tm1528)* mitotic germlines upon ionising irradiation.

Given that *clk-2* mutations lead to a DNA damage checkpoint dependent delay of embryonic cell cycle progression (Figure 2B) and given that *clk-2 (mn159)* germ cells showed elevated levels of RAD-51 foci indicative of faulty replication when shifted to the restrictive temperature at the L4 stage [22], we suspected that the germ cell cycle arrest of the *clk-2 (tm1528)* mutant might be due to the activation of the DNA damage checkpoint. We therefore examined if RAD-51 foci occur in the mitotic compartment of *clk-2 (tm1528)* germ cells. To our surprise we found that like in wild type germ cells, RAD-51 was mainly localized in the cytoplasm of *clk-2 (tm1528)* germ cells and did not form nuclear foci (Figure 6A, Video S4, Table 1). In contrast, *clk-2 (mn159)* shifted to the restrictive temperature of 25°C at the L1 or the L4 stage accumulated RAD-51 foci (Figure 6A, Video S6, Table 1) while *clk-2 (qm37)* formed fewer foci (Video S5, Table 1). Thus RAD-51 foci accumulate mostly in the weak *clk-2 (mn159)* allele as reported previously [22], while less foci formation is observed in *clk-2 (qm37)* and only very few RAD-51 foci can be found in *clk-2 (tm1528)* (Table 1).

**Table 1:** Quantification of RAD-51 foci in wild type and *clk-2* germlines.

Genotype	Temperature raised at	Number of RAD-51 foci/100 nuclei
wild type	20°C	2+/−1
*clk-2 (mn159)*	20°C	5.7+/−2.5
*clk-2 (qm37)*	20°C	4.5+/−1.8
*clk-2 (tm1528)*	20°C	3.3+/−2.8
*clk-2 (mn159)*	25°C shifted as L1	106.1+/−30.1
*clk-2 (qm37)*	25°C shifted as L1	24.3+/−13.6
*clk-2 (mn159)*	25°C shifted as L4	43.1+/−13.6
*clk-2 (qm37)*	25°C shifted as L4	20.0+/−8.0

Errors represent SD, for each genotype and condition tested 20 mitotic germ cells close to the distal tip cells were counted in 10 animals each.

The defect in RAD-51 foci formation in *clk-2 (tm1528)* might be due to a cell cycle arrest outside of S-phase or due to a failure to process DNA double strand breaks, which is needed for RAD-51 focus formation. To test whether DNA double stand break processing is defective in *clk-2 (tm1528)* mutants we tested whether focus formation occurred after inducing DNA double strand breaks by exposing worms to ionizing irradiation. Irradiation-induced RAD-51 focus formation indicated that double strand break processing occurs normally in *clk-2 (tm1528)* worms (Figure 6B). Summing up, these results indicate that the *clk-2 (tm1528)* deletion does not lead to excessive DNA damage and that CLK-2 is not needed for DNA double strand break processing.

**Figure 7:**
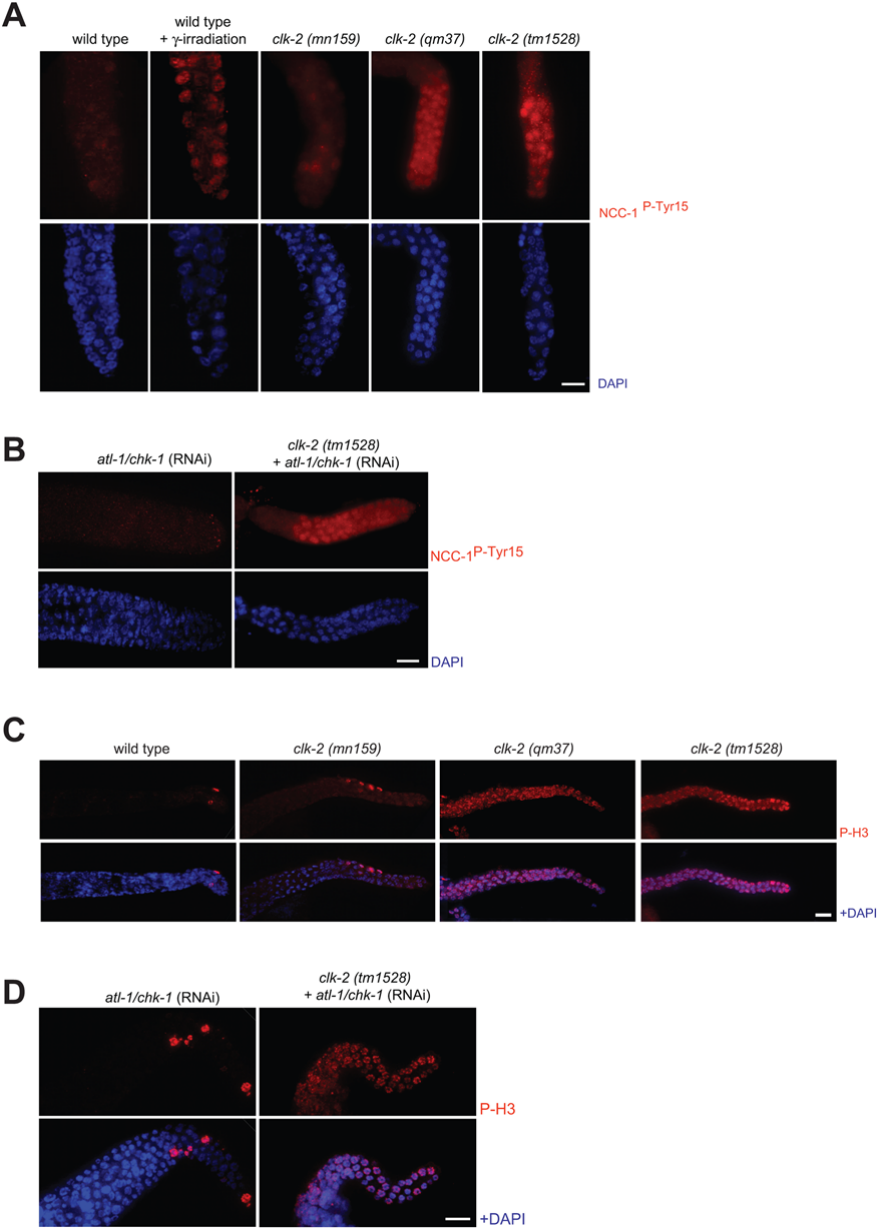
*clk-2 (tm1528)* and *clk-2 (qm37)* mitotic germ cells arrest in a distinct cell cycle stage. A) CDK-1/NCC-1 P-Tyr15 staining. Germlines of wild type, *clk-2 (tm1528)*, as well as *clk-2 (qm37)* and *clk-2 (mn159)* (shifted to 25°C at the L1 stage) were stained with NCC-1 P-Tyr15 antibody (red) and DAPI (blue). B) CDK-1/NCC-1 P-Tyr15 staining after *atl-1/chk-1* RNAi depletion in wild type and *clk-2 (tm1528)* worms C) PhosphoH3 staining. Germlines of wild type and *clk-2 (mn159*, *qm37* and *tm1528)*, propagated as described in A) were stained with phospho-H3 antibody (red) and DAPI (blue). D) Phospho-H3 staining of wild type and *clk-2 (tm1528)* depleted of *atl-1/chk-1* by RNAi. Efficiency of *atl-1/chk-1* RNAi depletion was confirmed by observing micronuclei and by scoring dead embryos in the next generation [22] (data not shown). For each representative picture shown in Figure 7 at least 15 germ lines were analysed.

To further analyse the cell cycle arrest associated with CLK-2 depletion, we asked if *clk-2 (tm1528)* germ cells arrest in a distinct cell cycle stage. To facilitate this analysis we first established G2 and M phase cell cycle markers. Prior to mitotic entry Cdk1 is kept inactive by Tyr-15 phosphorylation [34],[35]. An antibody recognizing Tyr-15 phosphorylation of mammalian Cdk1 cross reacts with the corresponding phospho-epitope of *C. elegans* NCC-1/CDK-1. Phospho-NCC-1/CDK-1 can be detected until late prophase in worm embryonic divisions [36]. To confirm that phospho-NCC-1 is indeed indicative of G2/M arrested germ cells, we irradiated wild type germlines and found that all germ cells arrested in G2 with high levels of NCC-1^P-Tyr15^ (Figure 7A). We observed NCC-1 Tyr-15 phosphorylation in only few wild type and *clk-2 (mn159)* mitotic germ cells but found that all *clk-2 (tm1528)* cells and *clk-2 (qm37)* cells showed high levels of NCC-1 Tyr-15 phosphorylation even in the absence of ionizing irradiation (Figure 7A).

The *clk-2* prophase arrest phenotype might be caused by a direct prophase defect or alternatively by replication defects which could trigger a checkpoint-dependent late G2/M cell cycle arrest. To assess these possibilities, we depleted *atl-1/chk-1* in *clk-2 (tm1528)* worms. The efficiency of *atl-1/chk-1* (RNAi) depletion was confirmed by observing germ cell micronuclei [22] and by the embryonic lethality of the progeny of RNAi depleted wild type worms (data not shown). We found that all cells of *clk-2 (tm1528) atl-1/chk-1* (RNAi) germlines were NCC-1 Tyr-15 phosphorylation-positive (Figure 7B). We therefore conclude that cell cycle arrest is unlikely to be mediated by activation of the ATL-1/CHK-1 DNA damage checkpoint (Figure 7B).

To further analyze the cell cycle stage of *clk-2 (tm1528)* germ cells we also used antibodies against phosphorylated histone H3 (P-H3). In *C. elegans* P-H3 staining can be observed in cells from prophase/early prometaphase to late telophase [37]. When wild type gonads were stained with anti-P-H3 antibody only 2–5 nuclei per gonad arm were stained and all stained cells displayed a metaphase-like morphology. While we observed the same phenotype for *clk-2 (mn159)* worms grown at 25°C, all germ cells were P-H3 positive in *clk-2 (qm37)* worms propagated at 25°C and in *clk-2 (tm1528)* worms (Figure 7C). However, P-H3 positive cells did not show a metaphase-like morphology. Rather, in most nuclei chromosomes appear to be partially condensed but not aligned at the metaphase plate suggesting a prophase or very early pro-metaphase arrest. This arrest neither depends on *atl-1/chk-1* (Figure 7D), nor on *cep-1* (Figure S4). Thus while *cep-1* and *atl-1/chk-1* are required for delaying cell cycle progression in *clk-2* embryos, the germ cell cycle arrest observed in *clk-2 (tm1528)* mutants does not depend on either of these genes.

**Figure 8:**
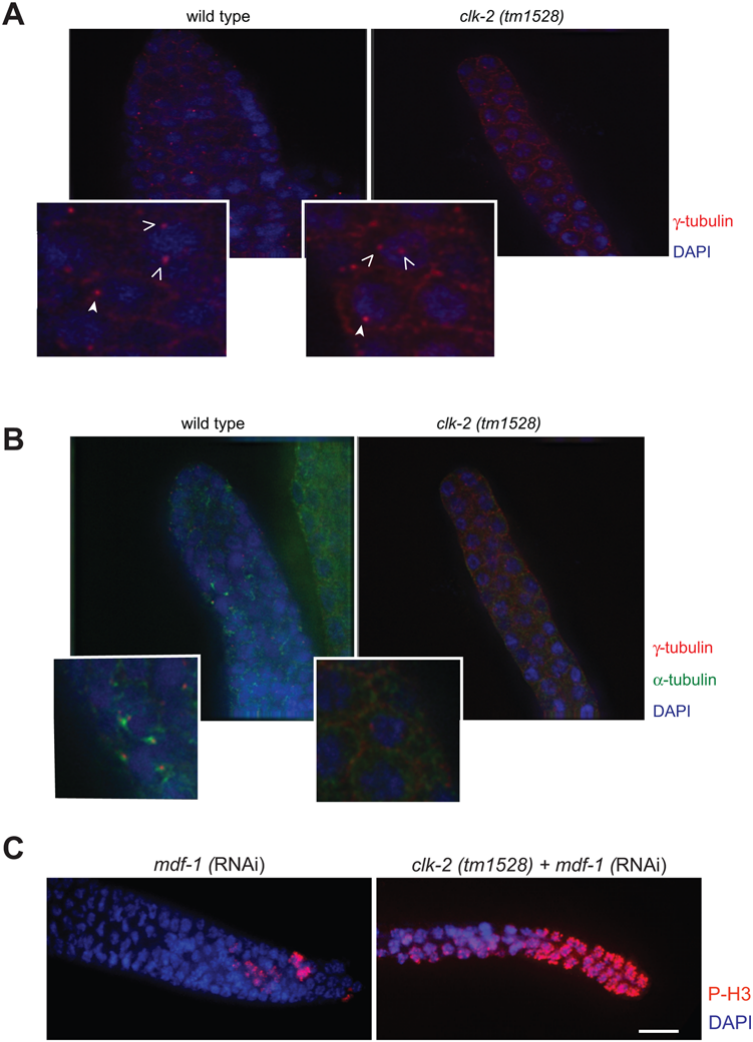
Centrosome duplication occurs but spindle formation is abolished in *clk-2 (tm1528)*. A) Images of fixed mitotic germlines of adult worms stained with γ-tubulin (red) and DAPI (blue). Arrowheads indicate centrosomes. Germ cells with one as well as two centrosomes can be found as indicated in the representative magnified panels. B) Germlines were stained for α-tubulin (green), and γ-tubulin (red) and DAPI (blue). In contrast to wild type, no mitotic spindles can be observed in *clk-2 (tm1528)* as indicated in the representative magnified panels. C) *mdf-1* (RNAi) does not abolish phospho-H3 staining (red) of *clk-2* germlines. *mdf-1* RNAi depletion was confirmed by the premeiotic like appearance of germ cells (data not shown) [41]. For each representative picture shown in Figure 8 at least 15 germ lines were analysed.

Given the early prophase arrest we also assessed centrosome behaviour in *clk-2 (tm1528)* germ cells. Centrosome duplication occurs during S-phase and centrosomes split during late G2 phase. In prophase, centrosome maturation is an essential prerequisite for the assembly of the mitotic spindle, and centrosomes can be visualized through the accumulation of α and γ-tubulin (for review see, [38]). Increased α-tubulin nucleation is followed by the formation of mitotic spindles [38]. When gonads were immunostained for γ-tubulin [39] to label centrosomes we found that centrosome duplication occurs normally in *clk-2 (tm1528)* worms (Figure 8A). Furthermore, double immunostaining for γ-tubulin and α-tubulin (Figure 8B) showed that several wild type germ cells exhibited accumulated α-tubulin, indicative of centrosome maturation and imminent spindle formation. In contrast, no α-tubulin accumulation and no spindle formation could be observed in *clk-2 (tm1528)* germ cells, although germ cells with duplicated and separated centrosomes were present (Figure 8B). These results raise the possibility that the prophase-like cell cycle arrest phenotype of *clk-2 (tm1528)* germ cells might be due to the activation of the spindle assembly checkpoint, which responds to defects in spindle formation and kinetochore-microtubule attachment and blocks anaphase progression until correct bi-orientation has occurred [40]. We therefore tested if the RNAi depletion of the *C. elegans* MAD1 spindle checkpoint gene ortholog *mdf-1* [41] rescues the cell cycle arrest phenotype observed in *clk-2 (tm1528)* worms. Even though both wild type and *clk-2 (tm1528)* strains displayed the typical previously described pre-meiotic like morphology of *mdf-1* (RNAi) germ cells [41] (Figure 8C), *mdf-1* (RNAi) *clk-2 (tm1528)* germ cells still uniformly stained P-H3 positive (Figure 8C). In summary, our analysis of *clk-2* germlines suggests that *clk-2* is essential for cell proliferation and that cells deficient in CLK-2 arrest in prophase without forming a mitotic spindle. The CLK-2 cell cycle arrest phenotype is independent of DNA damage and spindle checkpoint activation.

## Discussion

In aiming to define the developmental and cell cycle-related functions of *clk-2*, we found multiple roles of this conserved gene (summarized in Table 2). Aided by *C. elegans* cell lineage analysis, we found spindle orientation defects that can lead to cell fate transformations. Furthermore, dsDNA breaks accumulate in *clk-2* point mutations and embryonic cell cycle progression is retarded starting from the very first cell division. During early embryonic cell divisions the CLK-2 cell cycle delay can be rescued by depleting the ATL-1/CHK-1 pathway. CEP-1 accumulates in *clk-2* mutants and deletion of *cep-1* partially rescues the cell cycle delay associated with *clk-2* point mutations. Analysis of the *clk-2 (tm1528)* deletion reveals that these worms progress through embryogenesis (due to maternal rescue), but then halt cell cycle progression in the germline. This arrest phenotype, which occurs at an early prophase-like stage, appears to be independent of DNA damage and spindle checkpoint activation.

**Table 2:** Summary of *clk-2* phenotypes.

	*clk-2 (mn159)* 20°C	*clk-2 (mn159)* 25°C	*clk-2 (qm37)* 20°C	*clk-2 (qm37)* 25°C	*clk-2 (tm1528)*	*atl-1*
**DNA damage response signalling**	defective	defective	defective	defective	defective	defective
**RAD51 focus accumulation embryo**	wild type (nearly no foci)	elevated	wild type	elevated	n.d	n.d
**RAD51 focus accumulation germ line**	slightly elevated (nearly no foci)	elevated	slightly elevated	moderately elevated	wild type	elevated
**Embryonic cell cycle timing**	wild type	delayed	wild type	delayed	wild type	advanced
**ABar spindle orientation**	wild type	partially defective	wild type	partially defective	n.d	n.d
**Germ cell cycle progression**	wild type	wild type	wild type	defective	defective	defective

*clk-2* DNA damage signalling defects were described previously [5] and (Arno Alpi and Sandra Moser, unpublished observations). n.d. not documented but presumed to be wild type.

### *clk-2* Mutant Worms Do Not Phenocopy PIKK Depletion

It has recently been shown that CLK-2/TEL2 interacts with all PIKKs in budding and fission yeast as well as in mammals [8],[9],[42],[43]. CLK-2/TEL2 depletion leads to reduced levels of PIKKs, and using CLK-2/TEL2 mouse knockout lines it was shown that the half life of PIKKs is reduced in those cell lines [9]. This finding together with the notion that PIKK dependent checkpoint signalling is reduced in cells lacking CLK-2/TEL2 led to the hypothesis that CLK-2/TEL2 might function in checkpoint signalling by regulating PIKK kinase levels. Given the conservation of the CLK-2 PIKK interaction it is likely that this interaction also occurs in *C. elegans*, albeit we could not confirm this since we were unable to generate specific CLK-2 and ATR antibodies suitable for immunoprecipitation from worm extracts (data not shown). Nevertheless, our genetic results suggest that, at least in *C. elegans*, CLK-2 depletion does not phenocopy PIKK depletion phenotypes (summarized in Table 2). *atl-1*/ATR and *clk-2* mutations have opposite phenotypes during embryonic development and a *clk*-2 deletion, unlike *atl-1* depletion [22], does not lead to mitotic germ cell catastrophe. Concerning ATM, this worm PIKK is primarily involved in responding to UV-induced DNA damage where, like CLK-2 it is required for UV-induced apoptosis [44]. Furthermore, an *atm-1* deletion only shows weak defects in responding to ionizing irradiation [44], unlike *clk-2 (qm37)* and *clk-2 (mn159)* point mutations. Similarly, *clk-2* deleted worms do not resemble worms depleted for *tor-1*, which arrest in the L3 larval stage and show concomitant gonadal and intestinal degradation [45]. It is possible that partial *tor-1* depletion which results in a slow growth phenotype and enhanced longevity [46], overlaps with the *clk-2 (qm37)* phenotypes that include a slow growth and a relatively weak longevity phenotype [6],[7],[21]. However, the enhanced life span of *clk-2 (qm37)* worms is rather weak and *clk-2 (tm1528)* life span is dramatically reduced compared to wild type (data not shown). Our evidence suggesting that CLK-2/TEL2 might not predominately act by regulating PIKK stability is also supported by recent evidence from the budding yeast system. While steady state levels of the budding yeast ATR homologue TEL1 are somewhat reduced in *tel2-1* mutants, it was shown that TEL2 is required for the loading of TEL1 to sites of DNA damage [47]. In addition, the finding that TEL2 binding to the budding yeast MEC1 ATM like kinase is lost in *tel2-1* mutants while MEC-1 remains functionally intact [48], points towards the possibility that CLK-2 be able to regulate ATM and ATR PIKKs by mechanisms not directly related to TEL2 PIKK interaction.

### Is CLK-2 Required for DNA Replication in the Embryo?

We observed that cell cycle progression in early *clk-2* mutant embryos is generally delayed and is associated with DNA damage accumulation (for summary see Table 2). The *clk-2* cell cycle delay is partially suppressed by *atl-1/chk-1* and *cep-1* deletion. These results are surprising in the light of previous reports suggesting that CLK-2 and ATL-1 might act together in *C. elegans* DNA damage response signalling in germ cells [22]. These two proteins might thus act in different pathways during *C. elegans* embryogenesis. Our results suggest that ATL-1 is active in *clk-2* ts mutants. Thus even if there is a reduced level of ATL-1 protein in *clk-2* mutant worms, enough ATL-1 is left to cause a cell cycle delay.

In embryos depleted for DNA replication factors cell cycle progression is delayed starting from the very first cell cycle and upon division of the zygote the posterior daughter, referred to as the P1 cell, is particularly strongly affected [49]. This delay depends on the ATL-1/CHK-1 dependent DNA damage checkpoint. The relatively weak replication defect of CLK-2 could be due to partial loss of function in the *clk-2 (mn159)* or *(qm37)* point mutants or due to CLK-2 being required for faithful DNA replication rather than replication *per se*.

Our genetic analysis implicates the *C. elegans* p53-like gene *cep-1* in the cell cycle delay associated with *clk-2* mutants during embryonic cell divisions. Interestingly, deleting *cep-1* alleviates the cell cycle delay of *clk-2* mutants but does not have an effect on the delay caused by *div-1* mutants. Thus distinct DNA replication defects caused by *div-1* and *clk-2* depletion might lead to differential checkpoint activation. Our results implicate *cep-1* in an embryonic DNA integrity checkpoint. Future studies will be required to address how *cep-1* can slow embryonic cell cycle progression and which exact replication defects trigger CEP-1 accumulation.

### CLK-2 Is Required for *C. elegans* Germ Cell Cycle Progression

Despite a possible role of *clk-2* in embryonic DNA replication, *clk-2 (tm1528)* germ cells still undergo replication and do not display overt signs of genome instability. Analysis of *clk-2 (tm1528)* deletion mutants reveals that these worms progress through embryogenesis due to maternal rescue but then halt cell cycle progression in the germline. This arrest is distinct from the cell cycle arrest induced by DNA damage and does not require the ATL-1/CHK-1 DNA damage checkpoint and CEP-1. Similarly, this arrest does not require the spindle checkpoint. It will be interesting to assess if the cell cycle arrest is due to the requirement of *clk-2* in G2 cell cycle progression or due to the activation of a further checkpoint such as the p38 stress activated checkpoint [50]. *clk-2 (tm1528)* worms arrest in a phospho-histone H3 positive pro-metaphase like stage with partially condensed chromosomes, while DNA damage leads to a G2 arrest characterized by high levels of phosphorylated CDK-1 Tyr 15 and the absence of phosphorylated histone H3 in wild type worms. Interestingly, CDK-1 Tyr 15 is still phosphorylated in these arrested germ cells, indicating that these cells arrest with low CDK-1 activity. Thus our data suggest that there might be an uncoupling of mitotic events in *clk-2 (tm1528)* germ cells. Clk2/Tel2 has also been shown to be required for cellular proliferation in mouse embryonic fibroblasts. The arrest after CLK-2/TEL2 depletion is not uniform in TEL2 deficient MEFs. These cells arrest with an increased proportion of cells with a 2N or 4N DNA content, and a reduced S and M phase index, and were reported to show a ‘senescence-like flattened morphology’ [8]. Thus CLK-2/TEL2 might have additional functions in mammalian cells that are not directly related to cell cycle regulation. Alternatively, a cell cycle regulatory function of CLK-2/TEL2 might not be uniformly needed in all cell types.

Our analysis of *clk-2* mutant phenotypes reveals distinct CLK-2 functions in embryonic cell cycle progression and in germ cell cycle progression. The *clk-2 (tm1528)* null allele results in the most severe germline phenotype. At present we do not know if *clk-2* ts alleles are completely inactive when shifted to the restrictive temperature during early embryonic cell cycle progression. Indeed, as is the case for the germ cell cycle arrest phenotype, a complete inhibition of *clk-2* might result into earlier or more severe defects during embryonic cell divisions potentially resembling the *clk-2 (tm1528)* germ cell cycle arrest phenotype. We extensively tried RNAi to completely inhibit *clk-2* during embryogenesis using both RNAi injection and feeding procedures but never found a phenotype stronger than the phenotype of either *clk-2 ts* allele propagated at 25°C (data not shown). *clk-2* RNAi injections did not result in any phenotype [5], and only the RNAi feeding construct introduced by the Nilsen laboratory worked for RNAi feeding. Only, when we analyzed *clk-2* mutants kept at 25.5°C combined with *clk-2* (RNAi) we observed more severe defects as seen in *clk-2 (qm37)* and *clk-2 (mn159)* mutants at the restrictive temperature. Under these conditions we observe a further delay of cell cycle progression (particularly in the P lineage) as compared to *clk-2 ts* mutants kept at the restrictive temperature (Figure S5). This delay appears as *atl-1* independent. ATL-1 dependence was, however, difficult if not almost impossible to study due to severely abnormal cell divisions (data not shown), that often resulted in cell divisions where only one daughter cell received an intact nucleus. Even without *atl-1* (RNAi) treatment, nuclei often appeared as disorganized and at times fragmented under DIC optics (Figure S5), but we never observed uniform defects starting from the very first cell cycle, further complicating a detailed analysis (data not shown). Obviously, these findings will raise the question as to how CLK-2 might affect early embryonic cell divisions, which will be the subject of further studies. These studies will however, require new *clk-2* alleles as we currently can not rule out the possibility of off target effects associated with *clk-2* RNAi that might unspecifically enhance *clk-2* mutant defects.

At present, we can only speculate if the developmental, cell cycle related and DNA damage response pathway defects associated with *clk-2* mutations are due to a single molecular defect. We, indeed, favour an alternative model according to which CLK-2 affects multiple molecular processes. Our analysis which is based on an allelic series of *clk-2* mutants with increasing strength clearly indentifies distinct functions associated with CLK-2 during embryonic and germ cell cycle progression as well as during embryonic development. It was recently shown that TEL2/CLK-2 belongs to the ARM repeat superfamily of structurally related proteins [47] (Alexander Schleiffer, personal communication). Tandem ARM repeats fold together into a superhelical fold to form a surface for protein–protein interactions (for review see, [51],[52]). ARM repeat proteins are structurally related to proteins containing tandem HEAT motifs [51]. The demonstrated interactions between Tel2/CLK-2 and the HEAT repeat containing PIKKs suggests that TEL2/CLK-2 might act as an adaptor protein that impinges on multiple signalling pathways besides PIKKs through ARM/HEAT domain mediated protein-protein interactions. Our dissection of CLK-2 phenotypes in *C. elegans* is likely to stimulate future studies in mammalian cells addressing developmental and cell cycle-related functions of CLK-2/TEL-2.

## Material and Methods

### Strains

*C. elegans* strains were maintained at 20°C unless otherwise stated as described [53]. The following strains were used: *clk-2 (mn159)* [5], *clk-2 (qm37)* [54], *cep-1 (lg12501)* [55], *gld-2 (q497) gld-1(q485)* (gift of Tim Schedl), *div-1 (or148)* [49], *clk-2 (tm1528)* was generated and kindly provided by Shohei Mitani. The *clk-2 (tm1528)* deletion strain was backcrossed 5 times to reduce background mutations and balanced with *hT2 [bli-4 (e937) q418] by crossing to JK2689 [pop-1 (q4645) dpy-5 (e61)/hT2 [bli-4 (e937) q418]* to generate TG56 *clk-2 (tm1528)/hT2 [bli-4 (e937) q418]*. Further strains used were TG58 *cep-1 (lg12501)*; *clk-2 (qm37)*, TG57 *cep-1 (lg12501)*; *clk-2 (mn159)*, TG59 *cep-1 (lg12501)*; *div-1 (or148)*, TG60 *gld-2 (q497) gld-1 (q485)/hT2 [bli-4 (e937) q418]*; *clk-2 (tm1528)/hT2 [bli-4 (e937) q418]*.

### RNAi Analysis

RNAi was performed by using the feeding procedure [56]. RNAi-expressing bacteria were seeded on NGM agar plates containing 3 mM IPTG and 50 µg/ml ampicillin, and worms were added as L4 larvae the following day. Animals were fed with bacteria carrying an empty L4440 feeding vector [57] or *atl-1*, *chk-1* [15] and *mdf-1* feeding (MRC geneservice) constructs. Phenotypes were observed in F1 animals. F1 animals in the L4 stage were placed onto RNAi plates. F2 embryos were analysed after 24 h of incubation, and F1 animals were analysed after 48 h to observe germline phenotypes.

### Germ Cell Counts

Worms at the indicated time post-L1 were stained by DAPI using the following procedure. Animals were transferred to 100 µl M9 buffer and washed 3× with M9 buffer and resuspended in 1 ml 96% ethanol containing DAPI (200 ng/ml) for 1 h and rehydrated with 1 ml M9 buffer for 1 h. Worms were transferred into 3 µl of mounting solution (90% glycerol, 20 mM Tris pH 8.0, 1 mg/ml p-phenylenediamine) and mounted on slides. Germ cells were identified by nuclear morphology according to DAPI staining.

### Immunostaining

For the antibody staining, one day post-L4 adult gonads (for *clk-2 (tm1528)* 48 h post L4) were dissected in EBT (25 mM HEPES pH 7.4, 0.118 M NaCl, 48 mM KCl, 2 mM CaCl_2_, 2 mM MgCl_2_, 0.1% Tween 20) on a slide coated with poly-lysine (Sigma) and freeze-cracked. The slides were transferred to −20°C cold methanol, for 5 minutes and washed three times in PBS for 10 minutes at RT. Slides were blocked for 30 minutes in 0.5% BSA in PBST (PBS, 0.05% Triton-X100) and incubated overnight at 4°C with the primary antibody (1/1000 in 3% BSA in PBST). The next day, the gonads were washed three times in PBST each for 5 minutes at RT and incubated with the secondary antibody for 1 hour at room temperature. Gonads were washed three times in PBST each for 10 minutes and mounted with 5 µl mounting solution containing 0.5 µg/ml DAPI. Antibodies were used at the following dilutions: anti-α-tubulin antibody DM1A (Sigma) was used at 1/200, anti-γ-tubulin 1/5000 (gift of Carrie Cowen, IMP Vienna), anti PH3 1/400 (Upstate), anti RAD-51 1/200 [25], anti-Cdk1 1/100 (pTyr15, Calbiochem). Secondary antibodies used were anti-rabbit cy3 and anti-mouse FITC (1/1000, Jackson).

### 4-D Microscopy, Lineage Analysis, and Cell Cycle Timing

Methods for 4D-microscopy were described in [13]. Modifications of the 4D-microscope system are described in [31]. Embryos were recorded at 25°C and stacks of 25 DIC-images, viewing different focal planes of the developing embryo, were taken every 35 sec. The 4D-recordings were analysed using the SIMI Biocell program (SIMI Reality Motion Systems, Unterschleissheim, Germany; http://www.simi.com) [31],[13]. Cell cycle timing was determined by measuring the time between the two mitotic divisions (completion of cytokinesis).

### Fluorescence Microscopy

Deltavision microscopy was used to examine germlines using either a 60× or a 100×, UPlanSApo objective (Olympus; NA 1.40), Soft-WoRx software (Applied Precision), and a CoolSnap HQ (Photometrics) OCD camera. Three-dimensional datasets were computationally deconvolved, and regions of interest were projected onto one dimension.

### Western Blotting

Protein samples were resolved by SDS-PAGE analysis and transferred to polyvinylidine difluoride membrane (PVDF, Millipore). Membranes were blocked in 5% powdered milk, diluted in PBS Tween, then probed with primary antibody diluted in blocking solution for 3 hours. Primary antibodies were anti-CEP-1 (1/100 [55] ) and anti-CLK-2 (1/1000 gift of S. Boulton). Antibody binding was detected using anti-rabbit or anti-goat IgG coupled to horse radish peroxidase (Jackson) and proteins were visualized using ECL (Amersham) and autoradiography.

## Supporting Information

Figure S1 A) *clk-2* gene structure and mutant alleles. B) Western blotting with a CLK-2 specific antibody failed to detect any protein in *clk-2 (tm1528)*. The asterisk indicates a non-specific band that cross reacted with the CLK-2 antibody. Protein extracts were prepared from adult *clk-2 (tm1528)* worms.
(1.81 MB EPS)

Figure S2 *cep-1 (lg12501)* partially suppresses the slow growth phenotype of *clk-2 (mn159)* and *clk-2 (qm37)*. Adults were allowed to lay embryos for 4 h and the relative proportion of animals in L2, L3 and L4 larval stages was determined after 56 h at 20°C (A) or 48 h at 23°C (B). Error bars represent SD and are derived from three independent measurements.
(1.37 MB EPS)

Figure S3 Germ cell proliferation is reduced in *clk-2 (qm37)* worms. Representative images of dissected germlines stained with DAPI. Worms were shifted to 25°C at the L4 stage and fixed and stained with DAPI at the indicated time points.
(6.59 MB EPS)

Figure S4 CEP-1 does not affect the germ cell cycle arrest associated with *clk-2* mutations. Germlines of *cep-1 (lg12501)*, *cep-1 (lg12501)*; *clk-2 (qm37)* and *cep-1 (lg 12501)*; *clk-2 (tm1528)* mutants were propagated as described in Figure 7A and were stained with phospho-H3 antibody (red) and DAPI (blue). Scale bar: 10 µm.
(2.70 MB EPS)

Figure S5 *clk-2* (RNAi) phenotypes in *clk-2 (mn159)* and *clk-2 (qm37)* mutants. A) representative pictures as described in Figure 1C. B) lineage analysis as described in Figure 1B.
(14.85 MB EPS)

Table S1 Cell cycle timing of all cell cycles recordings depicted in Figures 1 and 2. Errors represent SEM, n = 5.
(0.08 MB DOC)

Table S2 AB and P1 cell cycle timing of wild type and *clk*-2 mutant worms with and without *div-1* (RNAi) treatment. S-phase length was determined as the period of time between cytokinesis and nuclear envelope breakdown as described [15]. M-phase length was determined as the time between nuclear envelope breakdown and cytokinesis as described [15]. Errors represent SEM, n = 6.
(0.03 MB DOC)

Video S1 Video depicting early embryonic development of wild type embryo shown in Figure 4.
(0.44 MB MPG)

Video S2 Video depicting early embryonic development *clk-2 (mn159)* embryo shown in Figure 4.
(0.43 MB MPG)

Video S3 Video depicting early embryonic development *clk-2 (qm37)* embryo shown in Figure 4.
(0.56 MB MPG)

Video S4 Video scanning through Z-stacks of representative picture *clk-2 (tm1528)* shown in Figure 6.
(0.20 MB MPG)

Video S5 Video scanning through Z-stacks of representative picture *clk-2 (qm37)* shown in Figure 6.
(0.25 MB MPG)

Video S6 Video scanning through Z-stacks of representative picture *clk-2 (mn159)* shown in Figure 6.
(0.31 MB MPG)
