# Correction: Functional Dissection of *Caenorhabditis elegans* CLK-2/TEL2 Cell Cycle Defects during Embryogenesis and Germline Development

**DOI:** 10.1371/annotation/5c0706a8-a9c2-452a-b061-a7c60dbc0499

**Published:** 2011-05-23

**Authors:** Sandra C. Moser, Sophie von Elsner, Ingo Büssing, Arno Alpi, Ralf Schnabel, Anton Gartner

The following information was omitted from the funding statement: "This work was partially funded by the Wellcome Trust, which also provided the fees for publication."

