# Supplementary material for: Functional Dissection of Caenorhabditis elegans CLK-2/TEL2 Cell Cycle Defects during Embryogenesis and Germline Development
Source: PLoS Genet. 2009 Apr 10;5(4):e1000451. doi: 10.1371/journal.pgen.1000451 (PMC2660272; doi:10.1371/journal.pgen.1000451)
Supplement: Table S1 — Cell cycle timing of all cell cycles recordings depicted in Figures 1 and 2. Errors represent SEM, n = 5. (0.08 MB DOC) [file pgen.1000451.s006.doc]

Table S1

|  | wildtype | *div-1 (or148)* | *clk-2 (mn159)* | *clk2 (qm37)* |
| --- | --- | --- | --- | --- |
| AB | 11.2+/-0.2 | 13.7+/-0.2 | 12.5+/-0.1 | 14.3+/-1.1 |
| P1 | 12.0+/-0.2 | 17.5+/-0.4 | 15.0+/-0.3 | 15.3+/-0.7 |
| ABa | 8.2+/-0.2 | 16.0+/-0.3 | 11.6+/-0.2 | 14.0+/-0.6 |
| ABp | 8.1+/-0.1 | 15.8+/-0.3 | 12.0+/-0.2 | 13.5+/-0.6 |
| EMS | 9. 5+/-0.2 | 18.4+/-0.3 | 13.8+/-0.3 | 15.5+/-0.3 |
| P2 | 10.9+/-0.3 | 20.9+/-0.3 | 18.4+/-0.3 | 18.9+/-0.2 |
| Abal | 12.2+/-0.3 | 19.1+/-0.3 | 14.2+/-0.3 | 14.6+/-0.5 |
| ABar | 12.7+/-0.3 | 18.6+/-0.3 | 13.6+/-0.3 | 14.3+/-0.4 |
| ABpl | 12.7+/-0.3 | 18.9+/-0.2 | 13.8+/-0.4 | 14.8+/-0.3 |
| ABpr | 12.9+/-0.3 | 18.7+/-0.3 | 13.6+/-0.3 | 14.9+/-0.2 |
| MS | 14.3+/-0.3 | 21.7+/-0.3 | 15.8+/-0.2 | 16.0+/-0.3 |
| EMS | 14.6+/-0.3 | 21.9+/-0.3 | 18.8+/-0.4 | 19.6+/-0.6 |
| C | 15.6+/-0.2 | 23.8+/-0.3 | 18.2+/-0.3 | 19.5+/-0.2 |
| P3 | 20.2+/-0.3 | 30.2+/-0.5 | 26.0+/-0.5 | 26.9+/-0.5 |

|  | *atl-1/chk-1N2* | *atl-1/chk-1*  *div-1 (or148)* | *clk-2 (mn159)* | *atl-1/chk-1*  *clk-2 (mn159)* | *clk2 (qm37)* | *atl-1/chk-1*  *clk-2 (qm37)* |
| --- | --- | --- | --- | --- | --- | --- |
| AB | 9.8+/-0.2 | 11.4+/-0.2 | 12.5+/-0.1 | 10.8+/-0.1 | 14.3+/-1.1 | 11.5+/-0.2 |
| P1 | 11.2+/-0.1 | 12.6+/-0.1 | 15.0+/-0.3 | 11.6+/-0.1 | 15.3+/-0.7 | 12.8+/-0.3 |
| ABa | 9.4+/-0.2 | 9.3+/-0.2 | 11.6+/-0.2 | 10.0+/-0.2 | 14.0+/-0.6 | 12.1+/-0.3 |
| ABp | 9.8+/-0.2 | 9.3+/-0.2 | 12.0+/-0.2 | 9.7+/-0.2 | 13.5+/-0.6 | 11.6+/-0.2 |
| EMS | 11.0+/-0.2 | 12.9+/-0.4 | 13.8+/-0.3 | 12.2+/-0.1 | 15.5+/-0.3 | 13.6+/-0.3 |
| P2 | 13.0+/-0.2 | 14.9+/-0.4 | 18.4+/-0.3 | 14.0+/-0.2 | 18.9+/-0.2 | 16.3+/-0.5 |
| Abal | 10.7+/-0.2 | 12.3+/-0.2 | 14.2+/-0.3 | 10.8+/-0.2 | 14.6+/-0.5 | 13.0+/-0.5 |
| ABar | 11.2+/-0.2 | 12.4+/-0.2 | 13.6+/-0.3 | 11.5+/-0.3 | 14.3+/-0.4 | 13.1+/-0.5 |
| ABpl | 10.5+/-0.2 | 13.9+/-0.2 | 13.8+/-0.4 | 11.3+/-0.2 | 14.8+/-0.3 | 14.3+/-0.6 |
| ABpr | 10.5+/-0.2 | 13.9+/-0.1 | 13.6+/-0.3 | 10.5+/-0.4 | 14.9+/-0.2 | 13.6+/-0.5 |
| MS | 12.0+/-0.2 | 13.0+/-0.2 | 15.8+/-0.2 | 11.6+/-0.1 | 16.0+/-0.3 | 13.4+/-0.3 |
| EMS | 13.0+/-0.1 | 15.3+/-0.1 | 18.8+/-0.4 | 13.4+/-0.2 | 19.6+/-0.6 | 14.4+/-0.3 |
| C | 13.3+/-0.3 | 14.9+/-0.2 | 18.2+/-0.3 | 14.8+/-0.1 | 19.5+/-0.2 | 15.6+/-0.3 |
| P3 | 19.7+/-0.3 | 20.4+/-0.2 | 26.0+/-0.5 | 21.6+/-0.2 | 26.9+/-0.5 | 22.5+/-0.6 |

|  | *cep-1 (lg12501)* | *cep-1 (lg12501); div-1 (or148)* | *clk-2 (mn159)* | *cep-1 (lg12501); clk-2 (mn159)* | *clk2 (qm37)* | *cep-1(lg12501);*  *clk-2 (qm37)* |
| --- | --- | --- | --- | --- | --- | --- |
| AB | 11.6+/-0.3 | 13.6+/-0.3 | 12.5+/-0.1 | 11.5+/-0.2 | 14.3+/-1.1 | 11.8+/-0.2 |
| P1 | 13.0+/-0.3 | 17.6+/-0.5 | 15.0+/-0.3 | 12.7+/-0.2 | 15.3+/-0.7 | 13.4+/-0.2 |
| ABa | 12.1+/-0.2 | 16.3+/-0.5 | 11.6+/-0.2 | 10.8+/-0.1 | 14.0+/-0.6 | 11.6+/-0.2 |
| ABp | 12.1+/-0.3 | 16.5+/-0.5 | 12.0+/-0.2 | 11.1+/-0.2 | 13.5+/-0.6 | 11.5+/-0.2 |
| EMS | 13.9+/-0.2 | 17.7+/-0.4 | 13.8+/-0.3 | 13.1+/-0.1 | 15.5+/-0.3 | 14.6+/-0.2 |
| P2 | 16.7+/-0.2 | 20.5+/-0.5 | 18.4+/-0.3 | 16.1+/-0.2 | 18.9+/-0.2 | 17.9+/-0.2 |
| Abal | 13.3+/-0.1 | 18.2+/-0.4 | 14.2+/-0.3 | 12.1+/-0.2 | 14.6+/-0.5 | 11.6+/-0.2 |
| ABar | 13.7+/-0.2 | 18.5+/-0.4 | 13.6+/-0.3 | 12.0+/-0.2 | 14.3+/-0.4 | 11.6+/-0.2 |
| ABpl | 13.9+/-0.1 | 18.3+/-0.5 | 13.8+/-0.4 | 12.1+/-0.2 | 14.8+/-0.3 | 11.5+/-0.2 |
| ABpr | 13.4+/-0.1 | 18.0+/-0.4 | 13.6+/-0.3 | 11.8+/-0.2 | 14.9+/-0.2 | 11.5+/-0.2 |
| MS | 15.1+/-0.2 | 21.0+/-0.4 | 15.8+/-0.2 | 14.5+/-0.2 | 16.0+/-0.3 | 14.6+/-0.1 |
| EMS | 16.3+/-0.2 | 21.5+/-0.5 | 18.8+/-0.4 | 17.4+/-0.3 | 19.6+/-0.6 | 14.6+/-0.1 |
| C | 16.7+/-0.3 | 22.8+/-0.5 | 18.2+/-0.3 | 16.6+/-0.6 | 19.5+/-0.2 | 17.9+/-0.2 |
| P3 | 22.7+/-0.2 | 31.2+/-0.7 | 26.0+/-0.5 | 23.6+/-0.3 | 26.9+/-0.5 | 17.9+/-0.2 |
