# Supplementary material for: Functional Dissection of Caenorhabditis elegans CLK-2/TEL2 Cell Cycle Defects during Embryogenesis and Germline Development
Source: PLoS Genet. 2009 Apr 10;5(4):e1000451. doi: 10.1371/journal.pgen.1000451 (PMC2660272; doi:10.1371/journal.pgen.1000451)
Supplement: Table S2 — AB and P1 cell cycle timing of wild type and clk-2 mutant worms with and without div-1 (RNAi) treatment. S-phase length was determined as the period of time between cytokinesis and nuclear envelope breakdown as described [15]. M-phase length was determined as the time between nuclear envelope breakdown and cytokinesis as described [15]. Errors represent SEM, n = 6. (0.03 MB DOC) [file pgen.1000451.s007.doc]

Table S2

|  | AB | | P1 | |
| --- | --- | --- | --- | --- |
|  | S | M | S | M |
| wild type | 11.0+/-0.1 | 4.0+/-0.2 | 12.8+/-0.3 | 3.8+/-0.4 |
| *div-1* (RNAi) | 11.8+/-0.6 | 3.4+/-0.3 | 17.1+/-0.8 | 3.5+/-0.3 |
| *clk-2(mn159)* *div-1* (RNAi) | 12.3+/-0.5 | 4.6+/-0.6 | 14.0+/-0.7 | 4.7+/-0.4 |
| *clk-2 (qm37)* *div-1* (RNAi) | 12.4+/-0.5 | 4.3+/-0.4 | 14.4+/-0.3 | 4.5+/-0.4 |
| *clk-2 (mn159)* | 11.1+/-0.3 | 3.9+/-0.2 | 12.7+/-0.3 | 3.9+/-0.2 |
| *clk-2 (qm37)* | 12.4+/-0.3 | 4.4+/-0.8 | 14.6+/-0.3 | 4.8+/-0.3 |
